# Spectrum of Clinical Research in Juvenile Idiopathic Arthritis: A Cross-Sectional Analysis of Registered Studies in Clinicaltrials.gov and Clinicaltrialsregister.eu

**DOI:** 10.3390/biomedicines9121860

**Published:** 2021-12-08

**Authors:** Ronny Lehmann, Markus Ries

**Affiliations:** 1 Center for Pediatric and Adolescent Medicine, Department of General Pediatrics I, Im Neuenheimer Feld 430, 69120 Heidelberg, Germany; ronny.lehmann@med.uni-heidelberg.de; 2 Center for Pediatric and Adolescent Medicine, Department of Pediatric Neurology and Metabolic Medicine, Im Neuenheimer Feld 430, 69120 Heidelberg, Germany; 3 Center for Rare Disorders, Heidelberg University Hospital, Im Neuenheimer Feld 430, 69120 Heidelberg, Germany; 4 Center for Virtual Patients, Medical Faculty, University of Heidelberg, Im Neuenheimer Feld 430, 69120 Heidelberg, Germany

**Keywords:** juvenile idiopathic arthritis, research registry, clinical trial, DMARD

## Abstract

The management of juvenile idiopathic arthritis (JIA) has improved tremendously in recent years due to the introduction of new drug therapies but remains complex in terms of non-pharmaceutical issues. In order to determine the direction of scientific progress by characterizing the current spectrum of ongoing clinical research in JIA, we analyzed all ongoing studies in the field of JIA—registered in clinicaltrials.gov and clinicaltrialsregister.eu—concerning sponsoring, enrollment, duration, localization, and particularly objectives. The close of the database was 7 January 2021. After identifying double-registered studies, *n* = 72 went into further analysis. Of these, 61.1% were academia-sponsored and 37.5% were sponsored by the pharma industry. The majority of the studies was of the interventional type (77.8%), while others (22.2%) were observational. The median planned enrollments were 100 participants (interventional studies) and 175 participants (observational studies), respectively. The duration differed remarkably from one month to more than 15 years, with a median of 42.5 months. A total of 61.1% of studies were located in a single country, and 38.9% were in several. Europe and North America clearly dominated the study localizations. The study objectives were DMARDs (56.9%), followed by diagnostics and disease activity measurement (18.1%), and medication other than DMARD (12.5%), besides others. Studies on DMARDs were mainly sponsored by industry, predominantly interventional studies on established and novel biologics, with several on specific issues such as systemic JIA and others. The spectrum of registered studies is currently centered on drug therapy and diagnostics, while other issues in JIA play a subordinated role in current research. Drug development was transferred from adult rheumatology into the JIA population with little innovation for children. Future research should take specific pediatric needs better into account.

## 1. Introduction

Juvenile idiopathic arthritis (JIA) is one of the most prevalent chronic diseases in childhood, with 16–150 cases per 100,000 population in developed countries [1]. Under-treated JIA results in joint corrosion, reduced quality of life and participation, and may cause persistent disabilities [2,3,4]. Furthermore, pediatric patients are at risk of local growth disturbances, (general) growth failure, and pubertal disorders [1,5]. JIA-associated uveitis and temporomandibular joint involvement are prevalent challenging treatment issues [2,6].

The management of JIA has tremendously improved over the last decades [7]. Molecular-immunology studies have led to a better understanding of etiologies and pathogeneses [3]. The introduction of biological and targeted synthetic disease modifying anti-rheumatic drugs (DMARDs) revolutionized the treatment and outcomes of JIA patients and will likely be applied in personalized treatment strategies [2,4]. Scientific research and drug development are brought into practice through the structural establishment of pediatric rheumatology networks and disease registers, and emerging guidelines for JIA [1,2,8,9,10,11,12].

Despite these considerable advancements, the treatment of JIA remains complex and improvable. Better definitions of disease entities and their pathogeneses are needed for improved classification and treatment strategies [1,8], as well as specific biomarkers for personalized treatment tuning [2,8,13,14]. Pediatric-approved DMARDs require long-term observation through registry studies [15], and recently approved DMARDs from adult medicine—i.e., in the treatment of rheumatoid arthritis—need to be explored for their potentials and risks in pediatric patients with JIA [16]. Novel drugs that selectively target the molecules or pathways involved in inflammation are needed to offer new treatment perspectives in refractory cases; therefore, prospective clinical studies are inevitable [2,8]. 

However, improving pediatric rheumatologic care is more than improving pediatric pharmacological care. A nontrivial question is: how can individual access to pediatric rheumatologic care be provided for children with such diseases [17,18]? Pediatric-specific issues (family-centered care, social integration and rehabilitation, etc.) must be addressed, as well as transition, as key issues of every chronic disease in childhood [19]. 

Against the background of JIA-associated and pediatric-specific health issues, as well as recent improvements in therapy and diagnostics, and known research needs, the purpose of this study is to characterize current clinical research in the field of JIA. 

## 2. Materials and Methods 

### 2.1. Search for Ongoing Clinical Studies

Web-based databases of the U.S. National Library of Medicine (clinicaltrials.gov) and the European Union Clinical Trials Register (clinicaltrialsregister.eu) were assessed on 7 January 2021 for ongoing clinical studies, with the search keywords “juvenile idiopathic arthritis” and the synonyms “JIA” and “juvenile chronic arthritis”. Filters were applied for age range (all age groups under 18 years) and study status. The term ‘ongoing’ refers to not yet finally completed studies at the time of analysis, including studies with the status “Recruiting”, “Not yet recruiting”, “Active/not recruiting”, “Enrolling by invitation”, “Suspended”, “Ongoing”, “Restarted”, and “Temporarily halted”, respectively. Data were downloaded for further analysis. 

STROBE criteria (Strengthening the Reporting of Observational studies in Epidemiology) were applied for the design, conduction, and reporting of this study [20].

### 2.2. Data Analysis

Microsoft Excel 2019 MSO, Edmond WA, USA, was used for data analysis. Standard techniques for descriptive statistics were applied. Study titles and description details were analyzed concerning sponsor, enrollment, duration, localization of study centers, and study type and objectives. Double-registered studies were identified and doublets were excluded. Missing data were not imputed. The sponsor was categorized into either industry or academia (including universities, public institutions and hospitals). Planned enrollment of participants was also extracted from description details. By the start date, ongoing “duration” of studies was calculated in months using the earlier date in the case of doublets in both registries. For localization of study centers, we displayed the top five locations for single- and multi-country studies, respectively, for which countries were clustered to their super-ordinated medical authorities (i.e., EU countries—EMA). Study details were analyzed for classification of interventional or observational studies, and their clinical phases, where appropriate. For the determination of study objectives, keywords were generated from study descriptions, and content analysis was used to determine answer categories [21]. 

## 3. Results 

### 3.1. Registered Studies

Overall, *n* = 56 studies registered on clinicaltrials.gov and *n* = 34 studies on clincialtrialsregister.eu met the search criteria. Of these, *n* = 18 studies were identified as being double-registered. The contents of *n* = 72 studies were further analyzed. In the following passage, we present the main results. For more details, see Supplementary File S1. 

### 3.2. General Findings

#### 3.2.1. Sponsor

Academia sponsored 44/72 (61.1%) of the found studies, and industry sponsored 27/72 (37.5%). One study was mixed sponsored. Of the academia-sponsored studies, 3/44 (6.8%) were doubled-registered in both registers, as were 15/27 (55.6%) of the industry-sponsored studies. 

#### 3.2.2. Planned Enrollment

For the interventional studies, the planned enrollment was median 100, with a minimum of 6 and a maximum of 340 participants. Planned enrollment for the observational studies was median 175, with a minimum of 10 and a maximum of 9000 participants.

#### 3.2.3. Duration of Studies

The start dates of *n* = 2 studies were given in the future at the time of assessment and were therefore not used for the calculation of duration. The duration of ongoing studies was calculated from *n* = 70 studies with a median of 42.5 months, a minimum of 1 month and a maximum of 183 months (more than 15 years). This means half of the ongoing studies started in late 2017 or thereafter. The start dates of the other half varied between 2005 and the first half of 2017. 

#### 3.2.4. Locations

Of the analyzed studies, 44/72 (61.1%) were located in a single country, and 28/72 (38.9%) in multiple countries. The most frequent countries for a single location were: France (9/44), the Netherlands (6/44), the United States of America (6/44), Canada (5/44), China (3/44), and Italy (3/44). When multiple countries were involved, the most frequent countries were: EU countries (24/28), the United Kingdom (17/28), Russian Federation (14/28), Mexico (13/28), and the United States of America (13/28). Geographically, European countries were involved in 54/72 studies (75.0%), North American countries in 30/72 studies (41.7%), South American and Asian countries in 14/72 studies (19.4%) each, African countries in 8/72 studies (11.1%), and Australia and Oceanian countries in 6/72 studies (8.3%). 

#### 3.2.5. Study Types

The found study type was interventional in 56/72 (77.8%) and observational in 16/72 (22.2%) studies. A clinical phase was given in *N* = 44/56 interventional studies:

- Phase I: 5 studies,
- Phase II: 4 studies,
- Phases I+II: 3 studies,
- Phase III: 22 studies,
- Phase IV: 10 studies.

### 3.3. Study Objectives 

**Figure 1:**
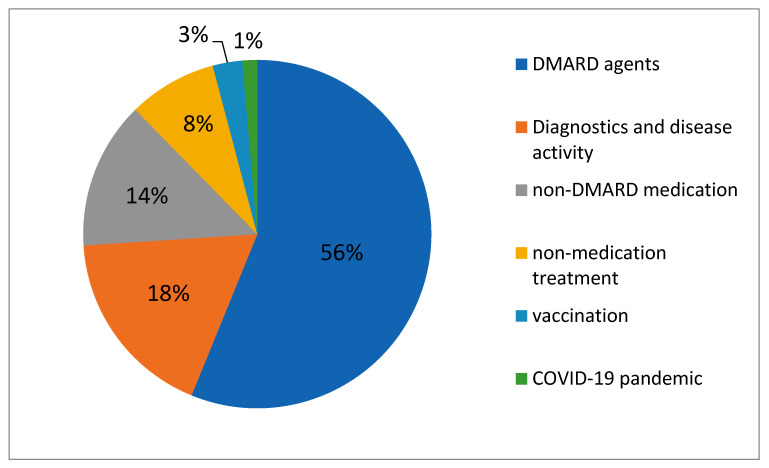
Proportions of study objectives.

For the proportions of study objectives, see also Figure 1. 

#### 3.3.1. DMARDs

**Figure 2:**
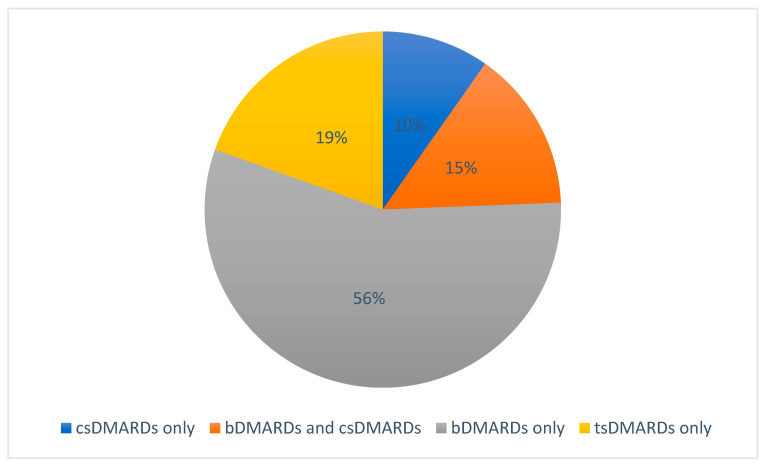
Proportions of DMARD agents involved in registered studies.

A total of 41/72 (56.9%) studies were related to DMARDs in the fields of JIA, including JIA-associated uveitis. Industry sponsors were involved in 27/41 studies. A total of 14/41 studies were sponsored by academia only. Studies addressed conventional synthetic DMARDs (csDMARDs; 10/41 studies), biological DMARDs (bDMARDs; 29/41 studies, hereof six studies also including csDMARDs), and targeted synthetic DMARDs (tsDMARDs; 8/41 studies). Conversely, 31/41 studies on DMARDs did not involve any csDMARD as a variable or control, and only 4/41 studies did not involve any bDMARD or tsDMARD, as seen in Figure 2. Biosimilar DMARDs were not involved.

Only 5/41 studies were of the observational type, while all others were interventional. The following DMARDs were specifically studied in these studies, in descending order (partly multiple agents involved per study):

- Methotrexate (MTX; 10/41 studies, hereof three observational studies),
- Abatacept (ABA; 6/41 studies, hereof one observational study),
- Etanercept (ETA; 6/41 studies),
- Tocilizumab (TOC; 6/41 studies),
- Adalimumab (ADA; 4/41 studies, hereof one observational study),
- Baricitinib (BAR; 4/41 studies),
- Tofacitinib (TOF; 3/41 studies),
- Canakinumab (CAN; 2/41 studies),
- Golimumab (GOL; 2/41 studies, hereof one observational study),
- Sarilumab (SAR; 2/41 studies),
- Secukinumab (SEC; 2/41 studies),
- Anakinra (ANA; 1/41 study),
- Certolizumab (CER; 1/41 study),
- Hydroxychloroquine (HCQ; 1/41 study),
- Ixekizumab (IXE; 1/41 study),
- Sulfasalazine (SUL; 1/41 study),
- Upadacitinib (UPA; 1/41 study).

**Figure 3:**
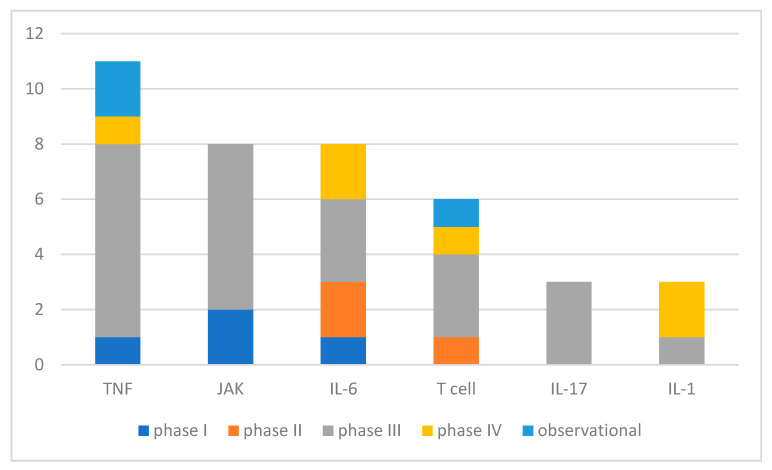
Number of studies concerning bDMARDs and tsDMARDs sorted for biological targets (interventional and observational studies, partly multiple agents involved per study). IL—interleukine, JAK—janus kinase, TNF—tumor necrosis factor.

Of the studied biological and targeted synthetic agents, the corresponding targets are shown in Figure 3. 

The enrollment of observational studies was median 833, with a minimum of 10 and a maximum of 9000. For clinical phases and planned enrollments in the interventional studies on DMARDs, see Table 1 and Table 2. All industry-sponsored studies were located in multiple countries, whereas only two of the academia-sponsored studies had locations in more than one country (the USA, UK, and several EU countries, respectively).

#### 3.3.2. Diagnostics and Measurement of Disease Activity

A total of 13/72 (18.1%) studies were related to diagnostics and disease activity in JIA in a broader sense. All of these studies were academia-sponsored and located in a single country. Studies concerned musculoskeletal and bone health, differential diagnosis of septic arthritis, imaging of arthritis, and temporomandibular involvement, as well as etiology and pathogenesis of systemic JIA, disease activity biomarker, and a national disease registry. The median enrollment of these studies was 90, with a minimum of 30 and a maximum of 1000. For more details, see Table 3.

#### 3.3.3. Medication Other Than DMARDs 

A total of 9/72 studies (12.5%) were related to medications other than DMARD and all of them of the interventional type, concerning the following medications: anti-IFN-gamma in systemic JIA, genicular nerve block, high-dose nicotinamide, dexmedetomidine, mesenchymal stromal cells, ondansetron, probiotics, recombinant interleukine-2, and triamcinolone hexacetonide. Except for the study on anti-IFN-gamma, studies were academia-sponsored and located in a single country. Median enrollment was 104, with a minimum of 6 and a maximum of 202. See Table 4 for more details. 

#### 3.3.4. Non-Medication Treatment

A total of 6/72 studies (8.3%) were related to non-medication treatment of JIA and of the interventional type. Four behavioral intervention studies concerned sleep and/or pain self-management of JIA patients, and a dietary intervention with specific carbohydrates was performed in one other, as well as a peer mentoring program for adolescents with JIA for acquisition of transition preparedness. All of these studies were academia-sponsored and located in a single country. Median enrollment was 30, with a minimum of 18 and a maximum of 262. See Table 5 for more details. 

#### 3.3.5. Vaccination 

A total of 2/72 studies (2.8%) were related to JIA and vaccination: one observational study concerned the frequency of the human papilloma virus vaccination among JIA patients (NCT04180228), and one interventional study concerned the safety and efficacy of a live attenuated measles, mumps, and rubella vaccine in JIA patients (EudraCT2007-001861-14). Both studies were academia-sponsored and located in France and the Netherlands, respectively. Enrollment was 150 and 280, respectively. 

#### 3.3.6. COVID-19 Pandemic

One observational, academia-sponsored study (1.4%) was related to the COVID-19 sanitary crisis and observed the impact on therapeutic management of JIA patients (NCT04407923). Localized in France, its enrollment was 150. 

## 4. Discussion 

Current clinical research in JIA is mainly focused on drug therapy—which predominantly means DMARD agents and sponsoring by the pharmaceutical industry—followed by studies on diagnostics and measurement of disease activity. Non-medication therapy and other issues were clearly secondary. In general, the size of interventional clinical studies was relatively small, with a maximum enrollment of 340 participants. The two main study locations were Europe and North America, followed by South America and Asia. 

### 4.1. Role of Sponsor in Clinical Studies

Ongoing registered clinical studies were sponsored by academia in about 60% of cases, and by (the pharma) industry in about 40%. Industry-sponsored studies were double-registered in both registries in slightly more than half of the cases, which is not common for academia-sponsored studies. 

Industry-sponsored studies almost exclusively studied DMARDs, except for one study that concerned treatment with anti-IFN gamma in systemic JIA. Only two of the industry-sponsored studies were observational (pharmacovigilance on MTX, ABA, ADA) while most others were interventional and tested DMARDs, namely BAR, CER, IXE, SAR, SEC, TOF, and UPA, for introduction into treatment of JIA. Typically, drugs had recently been labeled for rheumatic or chronic inflammatory bowel diseases in adults first, and use was then explored for JIA patients [16]. Only a few interventional studies sponsored by the industry concerned longer established DMARDs in JIA, TOC above all. All industry-sponsored studies had localizations in more than one country; we assume that this might be due to greater access to potential participants, as well as to potential pharma markets. 

Academia-sponsored studies did have much more varied objectives. Most of the observational studies (14/16) were conducted by academia, especially in the fields of diagnostics and disease activity measurement. In fact, at least one third of research in DMARDs is performed by academia, on longer established drugs including csDMARDs. Interestingly, withdrawal strategies in DMARD-treated patients play a significant role. Besides treatment with DMARDs, academia explores other possibilities of JIA treatment, including non-DMARD medications and non-medication (behavioral) treatment strategies. Multi-lateral localizations were an exceptional condition here; we assume that, in many cases, barriers between heterogenous legal areas increase the necessary effort for the realization of multilateral collaboration beyond feasibility for academia. 

### 4.2. Role of Novel DMARDs in Clinical Studies

Unsurprisingly, in clinical studies in the field of JIA, DMARDs are the big player. The introduction of biological and targeted synthetic DMARDs have tremendously changed the game up to today [2,4,7]. The longer goal of treatment is not only to improve complaints and disabilities, but also to achieve complete disease control for the best long-term outcome. The most frequent targets in DMARD treatment (by the count of registered studies and enrollments) are TNF, JAK, IL-6, and T cell. Of these, JAK inhibitors are the most recently introduced class of pharmaceutics to JIA. Baricitinib and Tofacitinib have comparable study numbers and enrollments in current research, as earlier introduced TNF inhibitors. In contrast, similar novel IL-17 inhibitors (IXE, SEC) are tested in distinctly fewer studies with smaller enrollments, which might be due to a limited application field in ERA and JPsA concerning JIA. Few novel agents were tested for targets with longer available bDMARDs, namely TNF (Certolizumab, phase III) and IL-6 inhibitors (Sarilumab, phase II). Furthermore, studies on IL-1 antagonist agents had a smaller part in DMARD studies, which are relevant only for systemic JIA in the field of JIA, besides other indications such as hereditary fever syndromes. 

Although the number of studies and total enrollments may be seen as markers for potential pharmacological candidates in general, clinical studies in children should limit the sample size needed and/or limit exposure to a placebo, such as through extrapolation of efficacy or early escape rules [22,23]. Rapidly expanding pharmacological options demand innovative clinical trial methods for accelerated authorization and introduction to JIA, and must not (only) rely on large sample sizes [24]. 

### 4.3. Targeting Specific Issues in JIA 

The number of current studies on specific pediatric issues—besides more pharmacological options—is low. Musculoskeletal impairment is addressed in only a few studies, while growth disturbances and pubertal disorders are not. Frequent prevalent issues in JIA, such as temporomandibular involvement and JIA-associated uveitis, were found to be specific objectives in only a few clinical studies in this study. Restrictively, this study did not search for uveitis-specific studies, as only studies that refer to the term ‘JIA’ were evaluated. Family-centered care, social integration, and rehabilitation, as well as transition were not found to be explicit issues in ongoing studies. Potential answers to the question of why these issues were not, or were scarcely, found in current studies are: a negation of research need where an improved arsenal of therapeutics is available; challenges in designing appropriate studies; or that the choice of two clinical trial registries for evaluation might not capture the bulk of non-medication trials. 

Most of the studies included several subgroups of JIA, mainly non-systemic forms or poly-/oligoarticular courses of JIA. Nevertheless, systemic JIA was specifically addressed in 12/72 studies (ten concerning DMARDs). Likewise, etiologically differing entities such as psoriatic and enthesitis-related arthritis were specifically addressed in 4/72 studies (all on DMARDs). 

### 4.4. Does Clinical Research Meet the Need for Research in JIA? 

It is not surprising that the majority (more than three quarters) of ongoing studies investigates particular treatment strategies on JIA. The value of scientific networking and collaboration, which brings research results into practice through guidelines and on-site rheumatologic care providers, can barely be shown by analyzing registered clinical studies. Specific issues of JIA, and pediatrics in general, were scarcely addressed. Transition into a vulnerable life stage is especially important for long-term outcomes, and of relevance to chronic diseases in pediatrics in general [25], particularly JIA, including somatic and mental health [19,26,27]. A direct reference to adolescents specifically, for instance, was only found in one of the studies, although in the context of peer mentoring. 

### 4.5. Limitations of This Analysis 

This study has several limitations. We used two registries (clinicaltrials.gov and clinicaltrialsregister.eu) whereby studies registered in smaller national registries, as well as from central registries outside Europe and North America, would be missing. Naturally, our study cannot determine studies and research that are not registered in any registry of clinical studies; this may be the case, especially for non-medication and/or observational studies. Our analyses rely on the accuracy of data input to these two registries. For the purpose of characterizing ongoing studies, we did not consider studies that were finally closed for further recruitment. Furthermore, we did not search for specific terms, i.e., uveitis, which may have revealed more research in these specific fields. 

This study did not formally analyze design and methodology of the clinical studies presented here, such as inclusion/exclusion criteria, study duration, endpoints and efficacy assessment. The European Medicines Agency has issued a “guideline on clinical investigation of medicinal products for the treatment of juvenile idiopathic arthritis”, which can be helpful for investigators [23]. In addition, innovative clinical trial methods and open dialogues between key stakeholders, such as investigators, regulators, and the pharmaceutical industry, may facilitate meaningful clinical research in the changing drug development landscape in JIA [24]. We consider this study a cross-sectional snapshot on the ongoing research in JIA in general, and not a specific in-depth exploration on research in predefined subsets.

## 5. Conclusions

While clinical research is mainly focused on drug therapy and diagnostics, other issues in JIA management are marginal topics in registered studies. Drug development was transferred from adult rheumatology into the JIA population with little innovation for children. Future research should take specific pediatric needs better into account.

## Figures and Tables

**Table 1:** Registered interventional studies involving csDMARD agents, clinical phases, and planned enrollments. * refers to a study involving multiple csDMARDs. sJIA—systemic JIA.

Agent	Phase	Registration Number	Enroll.	Age (Years)	Specifically Addresses	Start Year	bDMARDs Involved
MTX	II	EudraCT2008-006741-70	252	0–17		2008	n/a
	III	NCT02277444	130	2–17		2014	GOL
	III	NCT03728478	260	2–17	withdrawal	2019	ETA
	III	EudraCT2005-001086-34	200	2–17	withdrawal	2005	n/a
	IV	NCT03301883	74	2–17	sJIA	2018	TOC
	IV	EudraCT2013-003956-18	325	1–17	withdrawal	2014	ABA, ADA, ETA
	IV	EudraCT2014-003260-20 *	130	2–17		2016	n/a
HCQ	IV	EudraCT2014-003260-20 *	130	2–17		2016	n/a
SUL	IV	EudraCT2014-003260-20 *	130	2–17		2016	n/a

**Table 2:** Registered interventional studies involving bDMARD and tsDMARD agents, clinical phases, and planned enrollments. *, † refer to studies involving multiple bDMARDs. Start year in brackets refers to studies with start date in future of data acquisition. ERA—enthesitis-related arthritis, IL—interleukine, JAK—janus kinase, JPsA—juvenile psoriatic arthritis, sJIA—systemic JIA, TNF—tumor necrosis factor.

Target	Agent	Phase	Registration Number	Enroll.	Age (Years)	Specifically Addresses	Start Year
TNF	ADA	III	NCT02840175 *	62	2–17	withdrawal	2017
			NCT03816397	118	2–17	uveitis	2020
		IV	EudraCT2013-003956-18 †	325	1–17	withdrawal	2014
	ETA	I	NCT04585711	30	2–65		(2022)
		III	NCT01421069	109	2–30	ERA, JPsA	2011
			NCT02840175 *	62	2–17	withdrawal	2017
			NCT03728478	260	2–17	withdrawal	2019
			EudraCT2009-012520-84	100	2–17	ERA, JPsA	2009
		IV	EudraCT2013-003956-18†	325	1–17	withdrawal	2014
	CER	III	NCT01550003	193	2–17		2012
	GOL	III	NCT02277444	130	2–17		2014
JAK	BAR	III	NCT03773965	190	1–17		2019
			NCT03773978	197	2–17		2018
			NCT04088396	103	1–17	sJIA	2020
			NCT04088409	40	2–17	uveitis	2019
	TOF	I	EudraCT2011-004914-40	24	2–17		2012
		III	NCT01500551	340	2–18		2013
			NCT03000439	100	2–17	sJIA	2018
	UPA	I	NCT03725007	54	2–17		2019
IL-6	SAR	II	NCT02776735	100	2–17		2016
			NCT02991469	72	1–17	sJIA	2018
	TOC	I	NCT02165345	82	2–18	sJIA	2014
		III	NCT02840175 *	62	2–17	withdrawal	2017
			EudraCT2007-000872-18	108	2–17	sJIA	2008
			EudraCT2009-011593-15	185	2–17	withdrawal	2009
		IV	NCT03301883	74	2–17	sJIA	2018
			EudraCT2012-000444-10	43	2–17	sJIA	2012
T cell	ABA	I/II	NCT03733067	40	8–65		(2021)
		III	NCT01844518	187	2–17		2013
			NCT02840175 *	62	2–17		2017
			NCT03841357	306	2–16	withdrawal	2019
		IV	EudraCT2013-003956-18 †	325	1–17	withdrawal	2014
IL-17	IXE	III	NCT04527380	100	2–17	ERA, JPsA	2021
	SEC	III	NCT03769168	58	2–99	ERA, JPsA	2019
			EudraCT2016-003761-26	80	2–17	ERA, JPsA	2017
IL-1	ANA	IV	EudraCT2015-004393-16	55	2–17	sJIA	2016
	CAN	III	EudraCT2008-005476-27	122	2–99	sJIA	2009
		IV	EudraCT2018-004284-30	20	2–17	sJIA	2020

**Table 3:** Registered studies on diagnostics and measurement of disease activity.

Objective	Methods Used	Study Type	Registration Number	Enroll.	Age (Years)	Start Year
Physical activity	Indirect calorimetry	intervent.	NCT03913962	300	6–18	2019
Physical activity	Actigrafic measurement	intervent.	NCT04167488	50	4–18	2019
Physical activity	Exercise protocol	intervent.	NCT04671524	30	10–18	2020
Spine deformities	Trunk rotation measurement and radiological imaging	observ.	NCT04664231	200	4–16	2021
Differential diagnosis of septic arthritis	Proteic, cytokine and cellular markers	observ.	NCT03827759	90	0–14	2019
Differential diagnosis of septic arthritis	Mass spectrometry	observ.	NCT04460144	60	0–16	2020
Imaging	MRI	observ.	NCT02684695	200	12–24	2016
Imaging	Ultrasound	observ.	NCT04178837	33	1–17	2020
Temporomandibular involvement	Clinical examination, CT	observ.	NCT02218892	62	7–14	2011
Temporomandibular involvement, mouth and teeth health	DXA, MRI, caries scores	observ.	NCT03904459	452	4–18	2015
sJIA and adult-onset Stills disease	History, physical examination, laboratory including whole genome sequencing	observ.	NCT03510442	1000	0–99	2018
Disease activity biomarker	JADAS score correlation with (1–3)-β-D-Glucan serum levels	intervent.	NCT03984669	40	0–18	2019
National disease registry	Longitudinal data aquisition	observ.	NCT03245801	300	0–18	2017

**Table 4:** Registered studies on medication other than DMARDs.

Objective	Comments	Phase	Registration Number	Enroll.	Age (Years)	Start Year
Anti-IFN-gamma mAb	sJIA and MAS/sHLH	II	NCT03311854	10	0–18	2018
Genicular nerve block	Against triamcinolone	IV	NCT04687930	104	0–16	2020
High dose nicotinamide	Penetration in synovial fluid	I/II	EudraCT2018-002245-11	6	12–17	2019
Intranasal dexmedetomidine	Sedation for joint injections	IV	NCT03069638	109	1–18	2017
Mesenchymal stromal cells	In drug resistent JIA	I/II	EudraCT2012-002067-10	6	2–17	2014
Ondansetron	Premedication for MTX	n/a	NCT04169828	176	4–16	2019
Probiotics	Dietary supplementation	n/a	NCT03092427	120	1–7	2017
Recombinant IL-2	JIA and other rheumatic diseases	II	NCT04397107	46	0–17	2020
Triamcinolone hexacetonide	Personalised treatment	IV	NCT04614311	202	1–18	2020

**Table 5:** Registered studies on non-medication treatment.

Objective	Comments	Registration Number	Enroll.	Age (Years)	Start Year
Pain management	Behavorial intervention through yoga and aerobic dance	NCT03833609	25	13–18	2019
Pain and sleep management	Sleep manipulation protocol	NCT04133662	30	12–18	2018
Sleep management	Web-based program for parents	NCT04066205	60	8–13	2019
Sleep management for pre-schoolers	Web-based program for parents	NCT04354337	18	2–5	2020
Specific Carbohydrate Diet	As add-on therapy to low-medium disease activity	NCT04205500	30	0–16	2017
Transition preparedness	Peer mentorship through Skype calls	NCT03116763	262	12–18	2017

## Data Availability

The underlying data of this study are fully available as Supplementary Materials.

## Supplementary Materials

The following are available online at https://www.mdpi.com/article/10.3390/biomedicines9121860/s1. Supplementary File S1: Analyzed data (xlsx file).

## Abbreviations

ABA	abatacept
ADA	adalimumab
ANA	anakinra
BAR	baricitinib
bDMARD	biological DMARD
CAN	canakinumab
CER	certolizumab
csDMARD	conventional synthetic DMARD
DMARD	disease modifying anti-rheumatic drug
ERA	enthesitis related arthritis
ETA	etanercept
GOL	golimumab
HCQ	hydroxychloroquin
IFN	interferone
IL	interleukine
IXE	ixekizumab
JAK	janus kinase
JIA	juvenile idiopathic arthritis
JPsA	juvenile psoriatic arthritis
MAS/sHLH	macrophage activation syndrome/secondary haemophagocytic lymphohistiocytosis
MTX	methotrexate
n/a	not available
SAR	sarilumab
SEC	secukinumab
sJIA	systemic JIA
SUL	sulfasalazine
TNF	tumor necrosis factor
TOC	tocilizumab
TOF	tofacitinib
tsDMARD	targeted synthetic DMARD
UPA	upadacitinib
